# Publication in a medical student journal predicts short- and long-term academic success: a matched-cohort study

**DOI:** 10.1186/s12909-019-1704-x

**Published:** 2019-07-19

**Authors:** Ibrahim S. Al-Busaidi, Cameron I. Wells, Tim J. Wilkinson

**Affiliations:** 1 Department of General Medicine, Christchurch Hospital, Canterbury District Health Board, 2 Riccarton Avenue, Christchurch, 8011, New Zealand; 2 Department of Orthopaedics, Auckland District Health Board, Auckland, New Zealand; 3 Department of Medicine, Christchurch School of Medicine, University of Otago, Christchurch, New Zealand

**Keywords:** Academic medicine, Medical student, Medical student journal, Undergraduate research

## Abstract

**Background:**

Medical student journals play a critical role in promoting academic research and publishing amongst medical students, but their impact on students’ future academic achievements has not been examined. We aimed to evaluate the short- and long-term effects of publication in the New Zealand Medical Student Journal (NZMSJ) through examining rates of post-graduation publication, completion of higher academic degrees, and pursuing an academic career.

**Methods:**

Student-authored original research publications in the NZMSJ during the period 2004–2011 were retrospectively identified. Gender-, university- and graduation year-matched controls were identified from publicly available databases in a 2:1 ratio (two controls for each student authors). Date of graduation, current clinical scope of practice, completion of higher academic degrees, and attainment of an academic position for both groups were obtained from Google searches, New Zealand graduate databases, online lists of registered doctors in New Zealand and Australia, and author affiliation information from published articles. Pre- and post-graduation PubMed®-indexed publications were identified using standardised search criteria.

**Results:**

Fifty publications authored by 49 unique students were identified. The median follow-up period after graduation was 7.0 years (range 2–12 years). Compared with controls, student*-*authors were significantly more likely to publish in PubMed®-indexed journals (OR 3.09, *p* = 0.001), obtain a PhD (OR 9.21, *p* = 0.004) or any higher degree (OR 2.63, *p* = 0.007), and attain academic positions (OR 2.90, *p* = 0.047) following graduation.

**Conclusion:**

Publication in a medical student journal is associated with future academic achievement and contributes to develop a clinical academic workforce. Future work should aim to explore motivators and barriers associated with these findings.

## Background

Participation in research activities during medical school is associated with later academic success [1]. Early exposure to research enhances medical students’ research-related knowledge and skills, stimulates their interest in future involvement in research, and is associated with improved short- and long-term scientific productivity [1]. Multiple research training opportunities are available to medical students around the world, and a large proportion of medical students are interested in research careers [1, 2].

However, inexperienced medical students often face several barriers to publication in mainstream medical or scientific journals [3]. Unfortunately, this may discourage students from disseminating their research findings and considering a future career in academic medicine. To support students facing these challenges and foster the development of academic skills, more than 20 medical student journals (MSJs) have been established across the globe [4]. The main objective of MSJs is to promote and value academic research and publishing amongst medical students [2, 4]. MSJs provide a student-friendly environment where students can submit their work, develop research-related skills, and familiarise themselves with the peer-review process [2]. However, concerns often raised regarding the presumed low quality of published articles in MSJs; a recent analysis found most MSJs to have opaque peer-review policies and practices [5].

Despite the perceived importance of MSJs, their impact on future scholarly activities of medical students has not been evaluated [2, 6]. Furthermore, it is not known whether medical students who published original research articles in MSJs continue to be academically productive (e.g. completing higher academic degrees and obtaining academic positions) after graduation [2].

Many inter-related factors may contribute to long-term academic success, including early positive exposures to the publishing and peer-review process, development of key academic skills while still a medical student, inspiration to pursue a clinical academic career, and self-selection of students already interested in research. Essential academic skills such as manuscript writing and critical review are generally acquired through authentic experiential learning, and early exposure of medical students to the publishing process through MSJs may foster the development of these skills and contribute to the long-term success of aspiring clinical academics. The ‘student-friendly environment’ of MSJs may also support medical students during their early research careers and enable them to build confidence as they progress to larger and higher-impact projects.

The *New Zealand Medical Student Journal* (*NZMSJ*) is a student-run medical journal that publishes original (include research papers, review articles, and case reports) and non-original (feature/perspective articles, book/media reviews, and conference reports) contributions written by medical students from New Zealand. The journal employs a double-blind peer-review process undertaken by a combination of student and expert reviewers. The journal is indexed in Google Scholar, and has published over 300 articles since its launch in 2003 [6].

The aim of the present analysis was to evaluate the short- and long-term effects of publication in the *NZMSJ* on the scientific productivity of medical students, through examining the number of post-graduation publications in PubMed®-indexed journals, completion of higher academic degrees, and attainment of faculty rank after graduation.

## Methods

### Data collection

All articles authored by medical students in the *NZMSJ* from 2004 to 2011 (Issues 1–14) were retrospectively identified and analysed. The latter cut-off was chosen to allow time for students to graduate from the medical programme (6 years duration). Original research contributions to the *NZMSJ* (research articles, reviews, and case reports) authored by New Zealand medical students were identified by a hand search of the journal archives [7]. Other types of publications including editorials, feature/perspective articles, and book/media reviews were excluded from the analysis. Articles published by medical students from countries other than New Zealand were excluded. An article was considered to be student authored if the author biography clearly identified at least one student amongst the authors.

For each student author, the date of graduation from medical school was determined using publicly available New Zealand graduate databases [8, 9]. Gender-, university- and graduation year-matched controls were then identified from these databases in a 2:1 ratio (i.e. two controls for each student author) using a random number generator (Microsoft Excel for Mac, Version 15.41, Microsoft Corp., Redmond, WA, USA). Identified controls were manually cross-checked against journal archives to ensure these students had not published articles in the *NZMSJ* [7]*.*

PubMed®-indexed publications, for both cases and controls, before and after graduating from medical school were identified via searches conducted during the third week of October 2017 using student author name(s) and other identifiers such as country affiliation (New Zealand). Data regarding current clinical scope of practice, completion of higher academic degrees, and attainment of faculty rank were obtained from Google searches, New Zealand graduate qualification databases [8, 9], online lists of registered doctors in New Zealand and Australia [10, 11], and author affiliation information from published articles.

### Outcomes

The primary outcome was the number of PubMed®-indexed publications after graduation. Secondary outcomes were 1) attainment of university faculty positions, and 2) completion of higher academic degrees. Higher degrees were defined as any postgraduate degree obtained during (i.e. intercalated) or following medical school, and included Doctor of Philosophy (PhD), Masters, and Honours (e.g. Bachelor of Medical Science with Honours, BMedSc (Hons)) degrees, as well as postgraduate diplomas (PGDip) and certificates (PGCert). Data were primarily stratified according to student publication in the *NZMSJ*, though sub-analyses were also conducted stratifying individuals by gender.

### Statistical analysis

Collected data were entered into a pre-designed Excel spreadsheet. Descriptive statistics were used to summarise results. All continuous variables were determined to have non-parametric distributions using the Shapiro-Wilk test. Continuous variables were compared using the Mann-Whitney U test. Univariate odds ratios (OR) and associated 95% confidence intervals (CI) were calculated for each outcome using conditional logistic regression. Multivariable analysis was performed to control for confounding relationships between publication, attainment of higher degrees and faculty positions. A *p*-value <0.05 was considered statistically significant. Statistical analysis was performed using R (Version 3.5.2).

## Results

### NZMSJ student-authored publications

A total of 50 *NZMSJ* publications were identified (26 literature reviews, 22 original research articles, and 2 case reports), authored by 49 unique student authors. An additional seven articles were excluded from analysis as they were authored by overseas students.

Accounting for authors who published more than one article, there were 67 unique authors in total (49 students and 18 non-students). Almost all articles (*n* = 49, 98%) had only one student co-author, while only one article was authored by multiple students. Of the 49 unique student authors, 35 (71.4%) students entered the medical programme without a prior degree, 19 (38.8%) were female, and 30 (61.2%) were male. The majority of articles (67.3%) were authored by students in the second half of their degree (fourth to sixth year medical students). The median follow-up period after graduation was 7.0 years (range 2–12 years).

### PubMed®-indexed publications

Table 1 details the short- and long-term outcomes associated with student publication in the *NZMSJ*. One-third (32.7%) of *NZMSJ* authors identified had also co-authored at least one PubMed®-indexed publication prior to graduation (median 0 articles, range 0–23), compared with only 8.2% of students who had not published in the *NZMSJ* (Univariate OR 5.38, 95% CI 2.12–13.69, *p* < 0.001). NZMSJ authors also published a greater number of articles prior to graduation (median 0, mean 1.43, range 0–23 vs. median 0, mean 0.16, range 0–4, *p* = 0.01).

**Table 1:** Short- and long-term outcomes associated with student publication in the *NZMSJ*

Variable	Publication in NZMSJ, *N* = 49 (33.3%)	No publication in NZMSJ, *N* = 98 (66.7%)	*Univariate OR (95% CI)*	*P value*
Gender				1.000
Female	19 (38.8%)	38 (38.8%)	1.00 (0.50–2.02)	
PubMed®-indexed publication(s) prior to graduation				< 0.001*
Yes	16 (32.7%)	8 (8.2%)	5.38 (2.12–13.69)	
No	33 (67.3%)	90 (91.8%)		
PubMed®-indexed publication(s) following graduation				0.001*
Yes	30 (61.2%)	33 (33.7%)	3.09 (1.52–6.26)	
No	19 (38.8%)	65 (66.3%)		
Post-graduation higher academic degrees				
Any degree				0.007*
Yes	27 (55.1%)	31 (31.6%)	2.63 (1.30–5.32)	
No	22 (44.9%)	67 (68.4%)		
PhD				0.004*
Yes	8 (16.3%)	2 (2.0%)	9.21 (1.88–45.05)	
No	41 (83.7%)	96 (98.0%)		
Faculty position				0.047*
Yes	9 (18.4%)	7 (7.1%)	2.90 (1.01–8.30)	
No	40 (81.6%)	91 (92.9%)		

*Indicates statistically significant (*p* < 0.05)

Following graduation, this increased to 61.2% of NZMSJ authors versus 33.7% of controls (Univariate OR 3.09, 95% CI 1.52–6.26, *p* = 0.001). A similar relationship in the number of post-graduation publications was also found (median 1, mean 5.45, range 0–73 vs. median 0, mean 1.55, range 0–31, *p* < 0.001).

Only 14 *NZMSJ* authors (28.6%) had no pre- or post-graduation publications, compared with 57 (58.2%) of controls (Univariate OR 0.28, 95% CI 0.13–0.58, *p* < 0.001), while 8 *NZMSJ* authors (16.3%) had ten or more total publications, compared with 5 (5.1%) controls (Univariate OR 3.60, 95% CI 1.11–11.60, *p* = 0.03).

For the overall cohort, gender was not significantly associated with pre-graduation publication rates (18.9% males vs. 12.3% females, Univariate OR 1.66, 95% CI 0.60–4.28, *p* = 0.30). However, males had a greater number of post-graduation publications compared to female authors (median 1, mean 4.28, range 0–73 vs. median 0, mean 0.60, range 0–10, *p* < 0.001), and were significantly more likely to publish post-graduation (50.0% vs. 31.6%, Univariate OR 2.16, 95% CI 1.08–4.31, *p* = 0.03).

### Other postgraduate activities

At the time of data collection, 19 (38.8%) student authors had attained vocational registration in different clinical areas, 26 (53.1%) were residents/trainees in specialty training programmes, and 4 (8.2%) had ceased to practice medicine. Of the 98 controls, there were 44 specialists (44.9%), 46 trainees (47.0%), and 8 (8.2%) were no longer practicing medicine.

#### Higher degrees

Compared with controls, *NZMSJ* authors were significantly more likely to obtain any higher degree (55.1% (*n* = 27) vs. 31.6% (*n* = 31), Univariate OR 2.63, 95% CI 1.30–5.32, *p* = 0.007), including PhD (16.3% vs. 2.0%, Univariate OR 9.21, 95% 1.88–45.05, *p* = 0.004) following graduation. Of the 49 student authors, 16.3% (*n* = 8) had completed a PhD, 4.1% (*n* = 2) had completed a Master’s degree, 4.1% (*n* = 2) had completed a BMedSc (Hons), 34.7% (*n* = 17) had completed a PGDip, and 4.1% (n = 2) had completed a PGCert, while only 22 individuals (44.9%) did not have a higher post-graduate degree (percentages do not add to 100% due to individuals attaining multiple degrees).

Of the 98 controls, 2.0% (n = 2) had completed a PhD, 4.1% (*n* = 4) had completed a Master’s degree, 1% (*n* = 1) had completed a BMedSc (Hons), 21.4% (*n* = 21) had a PGDip, 7.1% (*n* = 7) had a PGCert, while 68.4% (*n* = 67) had no higher qualifications.

#### Faculty positions

Publication in the *NZMSJ* was significantly associated with higher attainment of academic faculty positions (Univariate OR 2.90, 95% CI 1.01–8.30, *p* = 0.047). Of the *NZMSJ* authors, nine individuals (18.4%) had attained a faculty position; one professor, one associate professor, four honorary/senior lecturers, and three junior academic staff. Seven controls (7.1%) attained a faculty role; none held professorial positions, while there were four honorary/senior lecturers, and three junior academic staff.

### Multivariable analysis

A multivariable conditional logistic regression model was constructed to adjust for the confounding effect of higher degree and faculty position attainment on post-graduate publication rates (*n* = 147) (Table 2). This identified that post-graduate publication was independently predicted by student publication in the *NZMSJ* (OR 2.64, 95% CI 1.23–5.68, *p* = 0.01), when adjusted for these factors. As expected, faculty appointment (OR 22.22, 95% CI 2.80–176.18, *p* = 0.003) was also highly predictive of publication, however attainment of a higher degree appeared to have no effect on post-graduation publication. All individuals who had completed a PhD had published at least one post-graduation article.

**Table 2:** Regression analysis results predicting post-graduation publication (*n* = 147)

Variable	OR	β	95% CI	*P* value
NZMSJ publication ^a^	2.64	0.38	1.23–5.68	0.01*
Higher degree	1.21	0.83	0.57–2.54	0.62
Faculty position	22.22	0.05	2.80–176.18	0.003*
Overall model fit; R^2^ = 0.18				

*Indicates statistically significant (*p* < 0.05)

^a^ As compared with gender, university- and graduation year-matched controls

## Discussion

To our knowledge, this is the first study to assess the short- and long-term impact of publication in a MSJ as medical student on future academic achievement. Findings from this study reveal that student publication in the *NZMSJ* is associated with higher rates of PubMed®-indexed publications, increased completion of higher academic degrees, and increased rates of appointment to faculty positions post-graduation.

The clinical academic workforce (individuals with training in both medicine and research) plays a critical role in bridging the gap between biomedical research and clinical practice [2]. However, recent reports from different countries indicate that the number of clinical academics has decreased or stagnated over the past few decades [1, 2, 12–14]. In addition to the several educational programmes and measures introduced by medical schools, findings from this study support the role of MSJs in developing academic skills amongst medical students and cultivating future clinical academics.

Multiple studies have shown medical student participation in research is associated with long term success in academia, including peer-reviewed publications, grants, and attainment of faculty positions [1, 15–17]. Indeed, a recent meta-analysis by Amgad et al. identified that students who participated in research during medical school were twice as likely to publish following graduation, and were over 6 times more likely to pursue an academic career [1]. The present analysis adds to this body of work, and is the first to demonstrate that publication in a MSJ as a medical student is associated with both short- and long-term academic success.

Previous research has demonstrated that medical students across the globe are interested in conducting research, and in pursuing academic careers [1, 18]. Furthermore, their work often results in a publishable product, with approximately 30% of medical student research resulting in a publication in the mainstream medical literature [1, 19, 20]. However, there is a considerable mismatch between the proportion of students’ reporting an interest in research and their actual participation in research, which have been reported as approximately 70 and 30% respectively [1]. Even for students who do participate in research, up to 70% of this work remains unpublished [1, 19, 20], representing an opportunity for interventions such as MSJs to support students to publish their work and develop their academic skills. Previous analyses have shown that a minority of student research publications (< 5%) are in MSJs compared to the mainstream literature [19, 20]. Surveys have identified that students often have a positive attitude towards publishing, but encounter several barriers, including perceived lack of research opportunities, not being supported to publish by their supervisors, not having the confidence or skills to write a paper, as well as time and financial constraints [2, 18, 21]. In addition to these common barriers, fear of rejection by journals is also a commonly cited reason by researchers for not publishing their work [22].

While publication in mainstream peer-reviewed journals is generally regarded as a key indicator of individual and institutional research productivity [1], MSJs have an important role in supporting students to publish their work and developing future clinical academics. The supportive student-friendly environment of MSJs likely encourages students to prepare and submit work that would otherwise not be published [3]. MSJs thus provide an important opportunity for students to attain and develop academic research skills at an early stage in their career, increasing the likelihood of subsequent research participation and pursuit of academic careers. However, other factors are also important in the development of academic clinicians; including clearly defined academic training pathways [13, 14, 23], and mentorship by senior researchers in supporting students [24].

Considerable attention has been given to the gender gap in academic medicine, with numerous published studies showing females are less likely to hold senior academic positions [12, 25], publish in mainstream medical journals [26], and to be appointed as editorial staff of journals [27, 28]. Just under 40% of NZMSJ student authors in this analysis were female, however we have recently shown that this gap has narrowed when including more current data [6]. Pre-graduation publication rates were not significantly influenced by gender, but analysis of post-publication graduation rates showed that males were significantly more likely to publish in PubMed®-indexed journals. These findings suggest that the gender gap in academic medicine develops and/or widens following graduation from medical school. Therefore, targeted strategies to address this gap may be most effective if introduced during medical school, or early following graduation, though this remains an area for future research.

Findings from this study need to be considered in light of certain limitations. The retrospective design of the study does limit data on other important variables including *NZMSJ* student authors’ participation in research activities during medical school and interest in research as a future career [29]. Furthermore, the use of a single MSJ, the *NZMSJ*, which predominately caters for medical students from New Zealand does limit the generalizability of the findings to other cohorts/MSJs. Despite the comprehensive search strategy which utilised multiple databases and online registries, it is possible that some data (e.g. completion of higher academic degrees and PubMed-indexed publications) were missed which may have affected the results of the study. The associations detected in this study may not necessarily be causal. The analysis included a relatively small, self-selected cohort of students who published in the *NZMSJ*. For example, students who are already interested in academic careers are more likely to be involved in research and motivated to publish their findings than their peers. This and other unmeasured potentially confounding variables could explain the associations observed in this study. Furthermore, students may have published in other international MSJs not included in this analysis, though based on the experience of the authors, this is extremely rare and unlikely to have influenced the results.

Future work should consider prospective assessment of students’ perceptions of MSJs, and their perceived impact on interest in research and academic careers. Prospectively measuring and controlling for the motivations of students to pursue academic careers may also be of value. Furthermore, several other MSJs exist around the world [4], and the findings of this study represent only a small cohort of authors from one MSJ in a single country. Replication of these findings in other independent cohorts is needed to validate and confirm the findings of the present study.

## Conclusion

Publication in *a MSJ* as a medical student is associated with increased publication in the mainstream medical literature pre- and post-graduation, increased completion of higher degrees, and higher attainment of academic faculty positions. These results suggest a novel strategy to further develop the clinical academic workforce; MSJs have an important role in developing academic skills amongst medical students and cultivating future clinical academics. Medical schools and funders of academic research should support the development and maintenance of MSJs financially and intellectually. Targeted interventions to reduce the gender gap in post-graduation publication rates may be most effective if introduced at the medical school level. Future work should aim to explore barriers and motivating factors.

## Data Availability

Subsets of data are available from the corresponding author on reasonable request.

## Abbreviations

BMedSc (Hons): Bachelor of Medical Science with Honours
MSJ: Medical student journal
NZMSJ: New Zealand Medical Student Journal
PGCert: Postgraduate certificates
PGDip: Postgraduate diplomas
PhD: Doctor of Philosophy
